# Designing a multifaceted survivorship care plan to meet the information and communication needs of breast cancer patients and their family physicians: results of a qualitative pilot study

**DOI:** 10.1186/1472-6947-13-76

**Published:** 2013-07-25

**Authors:** Rashida Haq, Lineke Heus, Natalie A Baker, Daisy Dastur, Fok-Han Leung, Eman Leung, Benjamin Li, Kathy Vu, Janet A Parsons

**Affiliations:** 1 Division of Hematology & Oncology, St. Michael’s Hospital, Toronto, ON, Canada; 2 Department of Medicine, University of Toronto, Toronto, ON, Canada; 3 Applied Health Research Centre, Li Ka Shing Knowledge Institute, St. Michael’s Hospital, 30 Bond St., Toronto, ON, Canada; 4 Canadian College of Naturopathic Medicine, Toronto, ON, Canada; 5 Oncology Clinical Research Group, St. Michael’s Hospital, Toronto, ON, Canada; 6 Department of Family Medicine, St. Michael’s Hospital, Toronto, ON, Canada; 7 Knowledge Translation Program, Li Ka Shing Knowledge Institute, St. Michael’s Hospital, Toronto, ON, Canada; 8 Department of Psychology, University of Toronto, Toronto, ON, Canada; 9 Cancer Care Ontario, Toronto, ON, Canada; 10 Leslie Dan Faculty of Pharmacy, University of Toronto, Toronto, ON, Canada; 11 Department of Physical Therapy and Graduate Department of Rehabilitation Science, University of Toronto, Toronto, ON, Canada

**Keywords:** Breast cancer, Survivorship, Qualitative methods, Pilot study, Survivorship care plan, Information needs

## Abstract

**Background:**

Following the completion of treatment and as they enter the follow-up phase, breast cancer patients (BCPs) often recount feeling ‘lost in transition’, and are left with many questions concerning how their ongoing care and monitoring for recurrence will be managed. Family physicians (FPs) also frequently report feeling ill-equipped to provide follow-up care to BCPs. In this three-phase qualitative pilot study we designed, implemented and evaluated a multi-faceted survivorship care plan (SCP) to address the information needs of BCPs at our facility and of their FPs.

**Methods:**

In Phase 1 focus groups and individual interviews were conducted with 35 participants from three stakeholder groups (BCPs, FPs and oncology specialist health care providers (OHCPs)), to identify specific information needs. An SCP was then designed based on these findings, consisting of both web-based and paper-based tools (Phase 2). For Phase 3, both sets of tools were subsequently evaluated via focus groups and interviews with 26 participants. Interviews and focus groups were audio taped, transcribed and content analysed for emergent themes and patterns.

**Results:**

In Phase 1 patients commented that web-based, paper-based and human resources components were desirable in any SCP. Patients did not focus exclusively on the post-treatment period, but instead spoke of evolving needs throughout their cancer journey. FPs indicated that any tools to support them must distill important information in a user-friendly format. In Phase 2, a pilot SCP was subsequently designed, consisting of both web-based and paper-based materials tailored specifically to the needs of BCPs as well as FPs. During Phase 3 (evaluation) BCPs indicated that the SCP was effective at addressing many of their needs, and offered suggestions for future improvements. Both patients and FPs found the pilot SCP to be an improvement from the previous standard of care. Patients perceived the quality of the BCP-FP relationship as integral to their comfort with FPs assuming follow-up responsibilities.

**Conclusions:**

This pilot multi-component SCP shows promise in addressing the information needs of BCPs and the FPs who care for them. Next steps include refinement of the different SCP components, further evaluation (including usability testing), and planning for more extensive implementation.

## Background

Breast cancer is the most common cancer in Canadian women affecting a large proportion of the population [1]. It is also the most common cancer in women globally, with the World Health Organization estimating 519,000 deaths from the disease in 2004 [2]. In Canada, there are approximately 153,000 breast cancer survivors, with an estimated 23,000 women receiving new diagnoses annually [1]. Due to improvements in screening and treatment, long-term survival rates for breast cancer are increasing, meaning that more women are living with a chronic condition. Once active treatment has been completed, they require ongoing follow-up and monitoring for disease recurrence, with many survivors experiencing ongoing effects of treatment [3,4]. The Institute of Medicine (IOM) recommends that survivorship care plans (SCPs) be employed with all cancer survivors, so that they not feel ‘lost in transition’ when they complete treatment and enter follow-up care [5]. However, recent research suggests that despite this recommendation, relatively few survivors receive SCPs and of these, most do not receive content in keeping with IOM recommendations [6,7]. A recent survey of National Cancer Institute-designated cancer centers revealed that SCPs are still not widely used with breast cancer patients (BCPs) [6].

Much of the literature on SCPs focuses on the provision of written treatment summaries and instructions on follow-up monitoring, and how these are delivered to primary care physicians from oncology specialists [8]. Most of these studies understandably emphasize outcomes such as recurrence rates and cancer-related mortality in relation to the model of follow-up care provided [8,9]. However more nuanced and contextually-mediated features of follow-up care and patients’ experiences of information provided have received less attention. A systematic review by Gagliardi and colleagues (2007) identified important research gaps related to health services delivery for breast cancer patients in Canada [10]. They noted that little research had been conducted regarding the information needs of BCPs during follow-up care and they specifically called for studies incorporating qualitative approaches to understanding service delivery, as well as the design of innovative interventions which specifically address the needs of BCPs [10]. Our qualitative pilot study addressed this gap directly, and focused on understanding and addressing the information *needs* surrounding ongoing supportive care for BCPs at our Canadian facility.

Our intent was to develop a pilot SCP relevant to the local context, while avoiding duplication of existing resources. When our study began, a number of ‘generic’ breast cancer SCPs had been developed for BCPs [11,12], however most of these focused more on documenting medical procedures completed as well as follow-up monitoring schedules, and routinely lacked information on a broader range of needs that might be of importance to patients (e.g. lists of locally available support services, information on psychosocial issues, etc.). We were not only interested in incorporating written summaries and instructions to guide ongoing care, but also in developing locally relevant web-based tools that could address a wide array of information needs of both BCPs and FPs in a shared-care model [8].

The study’s goals were: 1) to document information needs from the perspectives of BCPs, FPs and oncology specialist health care providers (OHCPs); 2) to design and implement an SCP based on identified needs; and 3) to evaluate the pilot SCP’s effectiveness at addressing these gaps.

## Methods

Communication within health care relationships is complex [13-17]. *What* is communicated, as well as *how* and *when* it is communicated are all important considerations when designing new modalities of information sharing. Qualitative methods are well-suited to understanding such complex phenomena, and to understanding how people receive and provide health care [18,19].

This single-centre pilot study employed an emergent and iterative design, with qualitative data collected before and after the development of a multi-component SCP. We first conducted an assessment of patient and FP information needs (Phase 1), and then designed and implemented a new SCP (Phase 2). Finally, in Phase 3 we evaluated the SCP to determine whether it met the needs of BCPs and FPs. The study received research ethics approval through the St. Michael’s Hospital Research Ethics Board. All participants provided written informed consent.

### Sampling and recruitment

Because the sampling and recruitment strategies were very similar for Phases 1 and 3, we offer a combined description here. A purposive sampling strategy was adopted and information-rich cases were sought [20], in order to provide an in-depth understanding of the experiences of BCPs and FPs. Participants were recruited from a single tertiary-care academic teaching hospital in downtown Toronto, Canada between March 2009 and November 2010. Inclusion criteria were willingness to engage in a focus group or in-depth interview, and the ability to understand and read English (the language of the SCP and in which interviews were conducted). BCPs who were either in follow-up care or about to transition to follow-up care were invited to participate. In keeping with the study’s qualitative methodology, BCP participants with a varied range of experiences were sought (e.g. patients receiving varied therapy regimens (adjuvant chemotherapy, radiation, hormonal therapy), age, stage at diagnosis, employment status).

For the provider participants, again we attempted to recruit FPs with varied experiences ( e.g. varying lengths of practice experience, age, men and women) and OHCPs representing a range of disciplines (e.g. surgeons, nurses, allied health practitioners). Primary inclusion criteria were that providers had to have some experience providing care to BCPs in their respective practices. For Phase 3, we attempted to link patients to their FPs. Our original intent was to evaluate the intervention when used by both patient and FP together but this did not prove feasible. As has been found elsewhere, recruiting FPs proved difficult [21]. All participants were volunteers and no incentives for participation were offered.

A total of 35 participants were recruited for the Phase 1 needs assessment (n = 21 BCPs, eight FPs and six OHCPs). Four focus groups were conducted, supplemented by nine individual interviews. Two focus groups were conducted with patients (n = 19) and one each with FPs (n = 6) and OHCPs (n = 5) respectively.

Following the development of the pilot SCP based on the Phase 1 findings, an additional 26 participants were recruited to provide feedback. BCPs recruited in Phase 3 were given access to the SCP tools over a period of 6 months prior to evaluating the tools. Two focus groups were conducted with BCPs, but this approach was not feasible for the FPs or OHCPs during this phase due to scheduling difficulties. For this reason, individual interviews were conducted with the provider participants. In all, 18 BCPs, five FPs and three OHCPs participated in Phase 3.

BCP participants had a median age at diagnosis of 55.5 years (range 35–85 years). One third were diagnosed with Stage 1 disease (53% were Stage 2), and 82% had invasive ductal carcinoma. Most patients (64%) were treated surgically with lumpectomy; 80% received radiation therapy and 74% underwent chemotherapy. Patient participant characteristics appear in Table 1.

**Table 1:** BCP participant demographics (n = 39)

**Characteristic**	**Frequency**
Median age at diagnosis	55.5 years (range: 35 – 85)
Cancer stage at diagnosis	
Stage 1	13 (33.3%)
Stage 2	21 (53.8%)
Stage 3	4 (10.3%)
Missing/unknown	1 (2.6%)
Cancer type	
Invasive ductal carcinoma	32 (82.0%)
Other	6 (15.4%)
Missing/unknown	1 ( 2.6%)
Surgery type	
Lumpectomy	25 (64.1%)
Mastectomy	13 (33.3)
Missing/unknown	1 (2.6%)
Received axillary lymph node dissection in addition to lumpectomy or mastectomy	14 (35.9%)
Number of patients receiving radiation therapy	31 (79.5%)
Therapy	
Chemotherapy alone	8 (20.5%)
Chemotherapy + endocrine therapy	21 (53.8%)
Endocrine therapy alone	9 (23.1%)
Missing/unknown	1 ( 2.6%)
Median duration of treatment	
(Chemotherapy and Biologic Therapy)	156 days (range: 117–577)
Proportion of patients receiving biologic therapy	6 (15.4%)

### Study procedures

#### Phase 1: Needs assessment

In Phase 1, focus groups and in-depth interviews were conducted to explore BCPs’ and FPs’ information needs. Focus groups were conducted according to participant profile (groups of BCPs, group of FPs, group of OHCPs). Interview guides were tailored to the specific stakeholder groups (BCPs, FPs or OHCPs). The topics covered in the Phase 1 interview guides are listed in Table 2. Patient participants were asked about their information needs, gaps in communication experienced during their care, their experiences of transitioning from active treatment to follow-up, their perceptions of their FP’s role in their follow-up care, and their preferred formats for receiving information. For FPs, they were asked about their own information needs, what is normally communicated to them regarding breast cancer follow-up by oncology staff, and the preferred formats for receiving information. OHCPs were asked to reflect on their own experiences of providing information to both BCPs and FPs and what they saw as the unique needs of both groups.

**Table 2:** Topics covered in interview guides

**Study phase**	**Purpose**	Topics covered*
***Phase 1***	Needs assessment	1) Experiences of information sharing at various points in their /their patients’ cancer care
		2) Information gaps
		3) Preferred formats for receiving information
		4) Perceptions of how information is shared between oncologists/OHCPs and FPs
		5) Provision of follow-up care to BCPs
***Phase 3***	Evaluation of SCP	1) Whether information needs were addressed effectively
		2) Any perceived gaps persisting
		3) Ease of navigation (web tools, paper-based tools)
		4) What worked well and what did not work well with specific components of SCP
		5) Information sharing between BCPs and FPs (and whether SCP tools facilitate this)
		6) Suggestions for improvement

*This list is not intended to be exhaustive of all topics covered, but rather is to highlight the primary topics for discussion.

#### Phase 2: SCP design

A pilot SCP was developed based on the Phase 1 findings, consisting of multiple components. The SCP consisted of two linked websites (one for patients, one for FPs). Each website was tailored to the specific needs identified by (a) patients and (b) providers. The patient site offered information and active links to a variety of resources, including local community support services, information about breast cancer and its treatment(s), common side effects of such treatments, and other supports, The FP site included a link to brief versions of the Canadian clinical practice guidelines for monitoring and follow-up of breast cancer, common side effects of treatment and their management, and a variety of locally available resources for their patients. The study team also conducted an environmental scan, compiling available resources of potential use to BCPs and FPs (many suggested by participants).

In addition to these web-based tools, a patient information booklet (or ‘passport’) was developed which included information about appointment bookings, key contact numbers and program personnel, etc. The information contained *some* of the same information on the website, but was not designed to duplicate the more extensive material offered by the web tools. The other paper tool developed was a clinical care plan (completed by the oncologist), which included a summary of treatment received to date, and an individualized follow-up/monitoring plan. This information was designed to be shared with both FP and patient. All components were developed to meet the needs of both BCPs and FPs within the context of our hospital’s breast cancer program and its surrounding local community.

#### Phase 3: Evaluation and feedback

In Phase 3, participants were asked to assess all components of the SCP and provide feedback via either focus groups or in-depth interviews. They were asked to reflect on the strengths and weaknesses of the various components, whether they thought the specific components addressed their respective information needs and whether they saw the SCP as facilitating communication between BCPs and FPs, or between OHCPs and FPs. Suggestions for improvement were invited. The topics covered in the Phase 3 interview guides are outlined in Table 2.

### Data analysis

Because the analytic approach was the same in Phase 1 and Phase 3 they are described together here. In keeping with the study’s qualitative methodology, data analysis occurred in conjunction with data collection [22]. An iterative and emergent design was employed, whereby preliminary findings were incorporated into subsequent data collection activities, and explored in depth. Audio tapes of focus group and individual interviews were transcribed verbatim. Two experienced qualitative analysts performed coding of written transcripts to identify emerging categories, themes and patterns [23,24]. NVivo software (version 8) was used to facilitate the comparison of themes across the focus group and interview transcripts as well as cross-coding to reveal inter-relationships among themes [13,17]. Comparisons of accounts pre- and post-intervention were also conducted. An approach of qualitative description was adopted, which entails an inductively derived thematic analysis [25,26]. Techniques to ensure analytic rigor included checking (e.g., triangulation, comparison of coding between analysts, seeking alternative explanations for the data) , questioning (e.g. interrogating the coherence of interpretation) and coding/data review by two experienced qualitative analysts [22]. In addition, the emerging analysis was reviewed and discussed with other members of the study team at intervals throughout the study, including a third individual with qualitative expertise.

## Results

### Phase 1: Needs assessment

Participants from all three stakeholder groups spoke about the dizzying array of ‘information’ generally available under the heading ‘breast cancer’ but that this deluge of facts and figures is typically overwhelming for patients as well as FPs. Moreover the timing and formats in which such information is shared lacks coordination and coherence, as experienced by the patients and FPs interviewed for this study. However the informational needs of patients differed from those of FPs. FPs told of needing basic information concerning follow-up monitoring in a user-friendly format. In contrast, patients told of wanting more detailed information tailored to their particular life stage and cancer type, and on a broad range of topics (addressing both medical and psychosocial concerns). We adopted a pragmatic approach to organizing the findings, whereby similarities and differences in perceived needs were compared and contrasted between the different stakeholder groups. Table 3 summarizes the primary themes and supporting quotes from the Phase 1.

**Table 3:** Phase 1 findings

**Group**	**Theme**	**Subcategory**	**Quotes**
Breast cancer patients (n =21 )	Type of information	Tailoring information to patient’s needs	“*One of the shortcomings is that there was very little preparation for any of the things that were coming. So there was a feeling that I scrambled a lot to understand whether what I was experiencing was completely normal, or whether it was something I should be paying attention to. And then figuring out if I needed to do something about that, or not.”* (PRIP3)
			*“ That’s a big thing too, is that the demographics of this population are changing. It’s a huge shift where we’re getting younger and younger and younger. … there wasn’t a lot of information for people below a certain age group.”* (PRFG1P4)
	Type of information	Importance of human resources (e.g. peer support)	“*I thought a peer counselling person to actually help someone through the process and through the challenges that exist would be a good idea.* “ (PRFG1P9)
			*“ … whether it’s a mentor in a hospital, whether it’s a nurse who at least is a specialist. But information isn’t good enough because it’s too overwhelming. And I think the right information at the right stage is the real challenge … but it isn’t just information.”* (PRFG1P7)
	Timing of information	Tailoring information to the patient’s needs (including stage of care)	*“Did you really give me the information when you did that, when you handed me a bag? Because you have to take in (to account) who the people are, and they are overwhelmed. So you need to give them the specific information in a timely way, and ensure they have the right information at the right time. I went home with a bag of information. There was no direction: here are some of the important themes you may want to pay attention to”* (PRIP3)
			*“ I think when you are diagnosed and you are going through treatment there are so many things coming at you … you have so much information that’s given to you to read that you need someone … almost a coordinator who comes into day care and you can walk through all of those things with someone…”* (PRFG2P4)
	Timing of information	Transitioning to follow-up care: questions about roles and responsibilities	*“I do have a concern about falling off the edge of the cliff, in the sense of, all of a sudden, do you just disappear? Will I have the same sense that my physical health is being monitored?”* (PRIP3)
	Relationship with FP		*“ In fact, throughout this whole process my family physician wasn’t involved at all … prior to this I was never really sick. So I didn’t see my family physician very often. So even to this day, I have more of a relationship probably at (oncology team)…”* (PRFG1P5)
			*“…in between all of my sessions, I would actually go and see him (family physician), just to bring him up to speed so he was part of the team … he and I have a fantastic relationship …he’s a really great GP. And I won’t give his name up! He’s amazing, and I think he’s helped me because of that.”* (PRFG1P6)
	Need for SCP		*“ …I think the challenge is for the environment to provide access to information and support around it”* (PRFG1P2)
Family physicians (N = 8)	Type and timing of information	Access to timely information	*“ … I think for me it’s the timeliness, so … I’m getting it two months down the road. So, more often I’ll have the patients come back and see me and I’ll be “Okay, what did the specialist say to you? What should we do, what’s the plan?” And I find I’m talking more to the patients instead of getting the reports or the information from the specialist. So, it would be nice even to get something preliminary you know, just the plan …”* (PRFGFP3)
	
			*“I guess I don’t have a huge number of, of breast cancer patients but one of the main issues again is about timelines . The information that I have received has arrived you know, months later and at that point it’s not very useful ….you don’t have the information on hand to talk to the patient when it’s, when it’s relevant.”* (PRFGFP2)
	Roles/ Responsibilities	Feeling ill-equipped to manage follow-up care	*“ We don’t always know the subt-, subtleties of the different findings and … what the implications are for prognosis and sometimes … it gets missed … but without us knowing it’s kind of, it makes things difficult, or it’s a lot of work for us to go digging into the pathology and the lymph nodes and trying to figure out what, what does that really translate into?”* (PRFGFP7)
			*“ Just give some kind of guidelines about what’s appropriate to monitor and … how long they should be on the medication, whether they need, it needs to be reassessed at some point by someone, the side effects of medications … but in the longer term, outside of that acute phase.”* (PRFGFP4)
			*“So I think in general, the biggest problem is that patients feel very lost after they’ve left. They’re very anxious that every symptom or everything (that) happens is related to their disease. And that family physicians are probably not well trained enough in this area and other areas of oncology that they don’t know how to decipher a lot of this either, and how to communicate that, and how to incorporate that into training or how to get them that support that they need for them to feel comfortable are the biggest issues.” *(PRIFP2)
	Relationship with FP		*“ when we mention to them possibly discharging them back to their family doctors, they feel that their family doctors have either not examined them throughout their treatment process, may not know what they’re finding, some still feel that their family doctor has missed the diagnosis, and that’s the biggest gap, they’re just not comfortable. And likewise, oftentimes we’ll get referrals back from old survivors who have minor problems that could be dealt with by the family doctor, but the family doctor is not comfortable dealing with it.”* (PRIFP2)
Specialist HCPs (n = 6)	Type and timing of information	Transitioning to follow-up care: suggested approaches	*So if you’re talking about survivors then and they finish their last chemo and then they say “Now what?” … if they could get something that they can take away with them, okay, follow-up every 3 months and …what other tests do they need and what(’s the) follow-up time? When do they see the medical oncologist? … Where does the family doctor fit into this? So, that if when a patient leaves after their final chemotherapy they have sort of a, a vision of …. how that’s going to look down the road, next year, next 2 years.* (PRFG1OHCP4)
	Roles/responsibilities	Sharing responsibility for follow-up care with FP	*“… but we’re not always sure if they (FPs) receive all the reports in a timely fashion. I think it’s the whole entire medical kind of system. Good news is no news … If everything is fine, then you’re not going to hear from me. Sometimes information is faxed, they may not get it in a timely fashion, sometimes things may get missed … I think we still have to follow up to make sure that the physician gets the report, so things don’t get missed.”* (PRIOHCP7)
	Need for SCP	Sharing information with BCPs	“ *I’ve had patients come to me and say ‘Well you know, so what do I do now?’ And they’ve already got (papers) and appointment books and other things and they just don’t even know it. And maybe they forget or they didn’t hear it or they’ve lost the little piece of paper … But maybe if we have something that they can take away with them….”* (PRFG1OHCP2)
		Importance of timely information sharing between OHCPs and FPs	“ *Having a really good system in the computer, so it’s easier to track and follow patients. …. and like a sheet that comes out and says that on such and such a date she (patient) was here for a mammogram. If all the information could just kind of appear about this individual….”* (PRIOHCP7)

### Patients needs

While BCPs felt well-supported in many ways prior to the SCP implementation, they identified important information needs that were not being met. Patients indicated that the *type of information* and the *timing of information* should be tailored to the individual’s unique context whenever possible.

#### Type of information required

Patients’ needs ranged from very practical and basic information (e.g. appointment scheduling, better understanding of follow-up monitoring) to in-depth information about their specific type of breast cancer. BCPs said that information provided should be reflective of the local (e.g. locally available support services) and national contexts (e.g. available health insurance, employment benefits, current clinical practice guidelines), and should be informed by international resources (e.g. the latest research). They also wanted information tailored and relevant to their age and life stage. For example, women in paid employment had different needs from those who were retired.

Prior to implementation we had initially envisioned a primarily web-based SCP. Both patients and OHCPs indicated that web-based interventions could assist patients to navigate through the wide array of information, resources, and support services currently available. However they also emphasized the importance of human resources (e.g. patient navigators, other clinical staff) as sources of support. BCPs suggested that some form of peer support program be implemented whereby other patients with similar characteristics (e.g. type of cancer, life stage) be provided. Both patients and OHCPs suggested a paper-based ‘passport’ or booklet be developed, which would provide an overview of the treatment process, and document specific treatments received. BCPs indicated that such a passport should be provided well before they transition out of treatment into follow-up care, and even as early as diagnosis/initiation of treatment.

#### Information timing

Patients recounted that receiving the appropriate information at the appropriate time was extremely important. When certain information is given too early, patients may feel overwhelmed and may not understand how it applies to them at that stage. Patients noted that our facility’s usual practice of providing a tote bag filled with pamphlets around the time of diagnosis (covering a range of topics, treatment stages, services) was not helpful to them. Conversely, participants reported that certain information was provided too late (e.g. information regarding the management of treatment side effects).

Interestingly, despite the study’s focus on the transition from active treatment to follow up care, BCPs were reluctant to concentrate solely on this period, and instead asserted that their information needs should be better addressed *throughout* their cancer journey. They depicted an evolving series of needs that stretched from diagnosis to follow-up monitoring, and anticipated that these needs would continue to evolve into the future (e.g. when and how to counsel their own daughters regarding their relative risk for future breast cancers). Nevertheless, they did characterize the transition to follow-up care as one of uncertainty and anxiety.

### Family physicians’ information needs

#### Feeling ill-equipped

FPs recounted that they frequently felt ill-equipped to provide follow up care and felt unsure of their role. FP participants reported that they need better access to information on caring for BCPs. FPs preferred paper-based formats for receiving patient-specific information regarding treatments and test results. They did not want a website that required a secure log in and password, but preferred a single-point-of-access website containing general information with pertinent links to brief versions of clinical practice guidelines, components of physical examination and monitoring, available resources/support services, lists of common treatments and their side effects (and management) and a few key articles. Any web-based intervention targeted to FPs should be user-friendly with quick links to practical resources for use during patient visits. FPs wanted printable patient information and checklists of topics to be addressed during follow-up visits.

### Phase 2: Developing the SCP

The SCP website component is divided into two linked sections, one for BCPs and one for FPs. The BCP guide contains information tailored to different phases of treatment (diagnosis, active treatment and follow-up). It also offers links to a range of resources available in the surrounding community. The FP guide has information tailored to physicians’ needs, including information about the side effects of various chemotherapy agents and their management, checklists for follow-up care and locally available services and resources for BCPs. The goal was not to duplicate existing resources, but instead to build on ‘best practices’ and resources currently available for both BCPs and FPs, by providing links via a single portal on the websites.

Because BCPs spoke at length about evolving needs throughout their cancer care journey during Phase 1, the various components within the SCP were designed to address needs encountered at different phases (from diagnosis, through active treatment, and into the follow-up period).

### Phase 3: Evaluation and feedback

Table 4 summarizes the post-implementation findings and offers supporting quotes.

**Table 4:** Phase 3 findings

**Group**	**Theme**	**Subcategory**	**Quotes**
Breast cancer patients (n = 18)	Comments on BCP website	“I wish I’d had this before”	*“I think there's a lot of good information there. And it would have been helpful to have it at the time of diagnosis.”* (POSFG1P7)
			*“I have been accessing information on the website very frequently. And I found it very comprehensive, very informative.”* (POSFG2P3)
			*“That bag of stuff was really overwhelming. I mean, you try, you pull it out and you looked at it. Then you put it in your cupboard. At least I did. This (website), it's so much easier. … You know specifically what you're looking for”* (POSFG2P7)
		Structure of site	*“Because when I was diagnosed, I was given the booklets, the pamphlets, the everything, just right here. You don't need that. You need a gradual step-by-step to where you can go back, like, to the website, and just refresh yourself as you journey along”*
			*“And I did find it extremely well-organized. I like the one page – every time you look something up it's kind of one page, you don't have to spend a lot of time scrolling through, looking for bits of information here and there.”* (POSFG2P4)
		Balance of amount and type of information (“Fear factor”)	*“I like this part of it where it's just brief, it doesn't get too in-depth because it would be, if you're just starting out, newly diagnosed … it would be too stressful…too stressful knowing too much … It might scare people, the fear factor might come in more.”* (POSFG1P2)
			*“I think it's very useful, especially the symptoms. But the thing is, I always have some fear when I go there because, it still reminds me, oh I used to be a patient.”* (POSFG1P6)
	Comments on patient booklet		*“ I think it's good because it's everything in a nutshell, instead of you getting these little business cards”* (POSIP2)
			*“Because when I was diagnosed, I was so overwhelmed that I couldn't even remember half of what had happened … to have something where you can write everything down.”* (POSFG1P3)
			“*It's very useful. All the useful information is there, right, starting from entering the hospital to where everything is located and what are the procedures, everything is very comprehensively explained.”* (POSFG2P6)
	Comments on paper-based SCP and Rx summary tool		*“I did give her the form. She didn't have any reaction, I didn't expect her to.”* (POSFG1P5)
			*“The other thing is, if you're seeing more than one doctor for any other illness, I just take it (summary and SCP) with me, and they're always very pleased to get it, and make a copy and put it in the file…. Whether it's my GP or whoever. Like, I see a couple of other doctors for different things, they were very pleased to have that.”* (POSFG2P7)
	Emphasizing health and wellness		*“So if you have something for healthy living, after this one … like a weekly menu or what you can take, how it helps you with your day-to-day life, and how if affects your body…”* (POSFG2P5)
	Relationship with FP		*“Actually I didn't see my family doctor until maybe 6 months late. I finished all the treatment. I went there for something else … I don't feel my family doctor contributed a lot, I mean, to this process.”* (POSFG2P6)
			*“…so I'm going to take the information to him, and talk to him and see if he's interested. He said you know, 'I wasn't part of your treatment, I wasn't part of the whole thing.' So really, I got the impression that well, ‘why do you want to involve me now,’ kind of attitude.”* (POSFG1P3)
Family physicians (N = 5)	Comments on FP website	“Here’s what I need to do, ABCD”	*“Okay, cognitive dysfunction, potential problems, so how do I identify it? And then what do I do next because here if I open this article, um, and this is interesting but it’s not like boom, boom, boom where do I go with you know, what do I do next, right?”* (POSIFP2)
			
		Suggestions for further improvement	*“(…) if we could print a symptom score and then just hand it out to the, hand it to the patient … oncologists (who) do that I think in their waiting rooms right? They have people fill out symptoms scores in the waiting room, you give it to the doctor and then we know ‘oh okay, this is how you’re doing right?’ So, it saves time because these questions are just as easily, in fact the symptom scores are validated in the way we ask questions randomly is not validated right? We’ll forget stuff, we don’t frame it in a way that necessarily is consistent and so …that’s something I think that would be helpful …”* (POSIFP1)
	Comments on paper-based SCP and Rx summary tool		*“So the concept is good … the follow up thing I liked, and then here’s the symptoms of recurrence. … stuff could jump out a bit more than it does … (you) might consider boxes for certain things, red flags [Um hmm], things to watch for and then you have like a box in red or something … but otherwise it’s good.”* (POSIFP1)
	Relationship with FP	On involving the FP in follow-up care: Transitioning	*“But that transition, that’s what makes the patient more comfortable you know, they, so they could be seeing the oncologist I don’t know every 3 months and then us maybe you know, every 6 months and then gradually they are seeing us every 3 months and the oncologist every 6 months and then just slowly fading out right? So that I think that would make it easier, it would make it easier for patients, it would make it easier for us. It’s just a question of timing it you know, right? But I, I haven’t seen that thus far right? Really now it’s kind of all or nothing, either we’re doing everything or good-bye right?”* (POSIFP4)
OHCPs (n = 3)	Feedback on website	General commentary	*“…I think you’ve got a lot of the major resources that people would need to get connected to, then the physicians themselves probably don’t know about either so I think it’s great that you’re replicating it on both sides. It’s looking good.”* (POSIOHCP3)
		Balance of amount and type of information (“Fear factor”)	*“ … do you tell everybody everything that might possibly happen or do you provide them that information once you’ve had the conversation in the context of the treatment they’re going to have? This is where people get scared…”* (POSIOHCP3)
			*“*‘*these could potentially occur, but will not necessarily happen to you, we will closely monitor you, and help you cope with possible side-effects.”* (POSIOHCP1)

### Patients

“I wish I’d had this before”

The initial aim of the SCP was to target BCPs who had completed active treatment and entered the follow-up phase. Although the patient participants had completed active treatment, they unanimously indicated that it would have been beneficial to have had these resources (i.e. website and booklet) at an earlier stage of care (i.e. at diagnosis, during active treatment). As a participant stated, “I wish I’d had this before.” While they liked much of the material presented within each of the SCP components, they also identified places where it could be improved. Overall however, the SCP’s components were perceived to provide more structured and tailored information than they had received prior to implementation.

#### Website

##### Layers of tailored information vs. a pile of brochures

Participants commented that the information available on the new website would have served their information needs much better than the “pile of brochures” they received when first diagnosed. In contrast to the brochures, participants recounted that the website offers information in ‘layers’ , rather than everything at once, facilitating navigation. They perceived these layers of information to be tailored to their needs, relative to their stage of care. They liked the appearance of the website, which they described as inviting, calm, friendly, and easy to navigate.

The support services/resource section was considered particularly helpful, directing BCPs to important information/community resources that had been lacking. This section was perceived as an important interface, connecting them to additional human resources in the community. Participants expressed that this resource section is a great first step to build upon, and anticipated that additional resources could be added. BCP participants also spoke at length about the need for striking a *balance between the amount and type of information.* They noted that too much information that is not contextualized (e.g. lists of possible symptoms of recurrence, or potential side effects) can foster feelings of anxiety and could deter patients from using the site.

#### Patient booklet (paper ‘passport’)

Participants appreciated the booklet as a practical tangible reference, that brings together important locally-relevant information (e.g. our hospital’s services, contact information, locations/map, appointments booking section), which was preferred over the separate appointment sheets received previously. Participants’ suggestions for improvement included instructions on how to make/cancel appointments, and a checklist of pre-appointment tips/reminders (e.g. not to eat prior to appointments, to prepare for long wait times, etc.).

#### Emphasizing health and wellness

Once their treatment was over, participants spoke of a desire to get on with their lives, and frequently characterized their cancer experience as a ‘closed chapter’. These participants expressed a strong resistance towards information and terminology that reminded them of having cancer, and being defined by the illness (i.e. ‘patient’ or ‘survivor’), when what they wanted was to be ‘normal’ again.

At first BCPs stated that they did not perceive a need for more information upon entering the follow up phase, mostly because they did not perceive themselves as still in need of cancer-related information. Nevertheless, the focus groups/interviews did reveal ongoing informational needs, ones identified as being of a less ‘clinical’ nature. They characterized these needs in terms of ‘health & wellness’, with a focus on being and staying healthy rather than illness. They offered suggestions that the website (and its title) should be framed ‘positively’ for patients in follow-up, focusing on lifestyle changes like nutrition and exercise advice. BCPs asked for more information about breast reconstruction, and a more supportive approach regarding symptoms of recurrence. Simply listing potential symptoms without any further explanation was not viewed as helpful and often caused participants to panic “about every little pain and symptom”.

### Family physicians

“Just tell me what to do: ABCD”

It proved difficult to recruit FP participants to evaluate the SCP materials. Nevertheless, 5 FPs were recruited who offered very positive feedback about the SCP, saying they appreciated having this type of resource, noting that they had “*bookmarked it for future reference”*. They found the website user-friendly, said the SCP would help them reduce their referrals back to specialists, and indicated that the tools would optimize the efficiency of clinic visits. Suggestions for website improvement included adding a ‘search’ function to enhance information retrieval and summaries of the most relevant information on a given page. Similarly, they suggested linking to abstracts rather than to full articles. FPs expressed a need for succinct instructions (“*Here’s what I need to do, ABCD*”) with less emphasis on in-depth information (*“I don’t need the pathophysiology”)*.

Printable checklists and validated patient-scored instruments were identified as the most helpful tools to add to future versions of the website (these were not included in the website’s first iteration). They foresaw BCPs completing these pre-appointment in the waiting room, to maximize the time available for clinic visits and prioritize components of follow-up care. Ease of use was a central concern for the FPs interviewed. Finally, FPs also commented that it is difficult to remain abreast of developments in different specialty areas, especially since FPs are inundated with practice guidelines in a myriad of specialty areas. Getting the attention of FPs regarding BCP survivorship concerns means competing with many other demands.

#### Paper-based care plan

Both patients and FPs found the paper-based care plan (treatment summary and follow-up care plan) an important tool for smoothing the transition between active treatment and follow up. Patients recounted that their FPs seemed more engaged in their care, and appreciated the team approach fostered by this three-way communication tool. The paper-based tools helped BCPs appreciate the FP role in follow-up care (e.g. monitoring between specialist visits, routine blood work) and increased their confidence in their FPs’ abilities to fulfill this role.

FPs appreciated having a succinct summary of treatment and ongoing concerns, rather than having to go through long reports, and expressed feeling better equipped to provide follow-up care. Some FPs felt that the purpose of the paper-based forms was not always clear. FPs as well as patients would prefer if the forms were sent directly to the FP (prior to patient visit), rather than through the patient themselves. However patients also liked having a copy for their own records.

### The transition to follow up care

“All of a sudden do you just disappear?”

In both the pre- and post-intervention interviews, BCPs depicted the transition period from active treatment to routine follow up as one characterized by fear and uncertainty (see Tables 3 and 4 for supporting quotes). The accounts of BCPs reflected ambivalence concerning treatment completion and relinquishing the relationships they had developed with specialists. They expressed both a sense of relief to ‘be done’ with treatment and hesitancy to leave specialist care and transfer follow-up duties to their FP. Patients asked for clearer explanations of the roles and responsibilities of the various health care providers involved (including the FP). Concerns over who would conduct their follow-up examinations and how closely they would be monitored were expressed. Patients described varying experiences of FP involvement in their care, however most reported that they had little interaction with their FPs following their diagnosis with breast cancer. This extended into the follow-up period, and most participants perceived their FPs to have little or no expertise in this area.

Both pre- and post-intervention, BCPs spoke at length about how their pre-existing relationship with their FPs affected their comfort level with returning to their FPs for follow-up care. If the relationship was uncomfortable, or if they perceived that their FP had missed the diagnosis, they were reluctant for their FP to assume responsibility for follow up. If they had a strong and comfortable relationship with their FP prior to cancer, they were more willing to return to their FP for follow-up. Regardless of the quality of their relationship with their FP, most patients perceived their oncologists to be the most trusted source of breast-cancer-related information.

## Discussion

This qualitative pilot study assessed the information needs of BCPs and FPs, which in turn informed the development of a new multi-component SCP. The SCP was designed not to duplicate existing resources, but instead sought to consolidate these into a single package. Based on the pre-implementation findings, we conceptualized a SCP that included multiple components: web-based and paper-based, and tailored to the specific needs of both patients and providers. This is a more holistic approach than is usually cited in the literature; most SCPs consist of providing written treatment summaries and instructions for follow-up monitoring [8,9,11]. Fortunately this is beginning to change and more tools are available than when we began our study [27,28]. However, our patient participants emphasized the importance of providing access to a variety of information resources from the hospital where they received treatment. Our evaluation indicates that all of our various pilot SCP components do indeed address many information needs previously identified, but that BCPs have additional concerns that remain to be addressed (e.g. more information on health and wellness, the quality of the pre-existing relationship with FP). A Canadian survey of cancer patients (including BCPs) revealed that BCPs were the least satisfied with primary care follow-up, but did not explore the underlying reasons in depth [3]. Grunfeld and Earle (2010) note that there is little research on patients’ perspectives concerning models of survivorship care [29], and our study begins to address this gap, by illuminating patients’ concerns about and experiences of primary care follow-up. An underlying assumption of our SCP’s design is that BCPs want to be actively engaged in their care throughout their cancer journey and that they are not passive recipients of information. This assumption was borne out by the accounts offered by the BCP participants in our study. In a recent systematic review, Howell and colleagues (2012) note that there is little research on how to actively engage survivors in their follow-up care [8], and again, our study begins to address this issue. Our participants portrayed a complex and evolving set of information needs, as well as a desire to interact with this information in ways that were comfortable (i.e. web- or paper-based, depending on the patient), manageable (i.e. not overwhelming and suitable to different stages of care), supportive (i.e. from a trusted source – namely their treatment facility), and sensitive to individual circumstances and preferences.

The information needs of patients and FPs were distinct. BCPs’ needs change throughout their cancer journey, but this does not necessarily follow a particular or set chronology -- from diagnosis and treatment to follow-up. Rather they spoke of needs that were fluid and evolving, based on their given context (e.g. younger women, older women), circumstances (e.g. parental, employment, and marital status, etc.), and disease profile (based on relative risk of recurrence, presence of metastatic disease, etc.). “One size does not fit all.” Information needs to be patient-centered and tailored to individual needs. Many survivorship studies emphasize issues related to follow-up monitoring, inter-professional communication of test results, and guideline adherence by FPs [9,30-33]. But it also raises the question of how ‘patient-centered’ many SCPs actually are. In our study, BCPs identified many additional topics that SCPs should address. The term ‘personalized medicine’ usually refers to new genetically-based treatments, but it should also apply to tools like SCPs. Other researchers have noted that addressing ‘non-oncologic’ patient concerns is central to holistic, patient-centered care [34].

The ability to capture features of the lived experiences of patients and the contexts in which they recover is one of the strengths of our study’s design. A recent Canadian RCT by Grunfeld and colleagues (2011) compared a standardized SCP versus usual standard of care and found no significant differences between the two arms [9]. However the authors acknowledge that the patient-reported outcome measures employed in this RCT may not have been sufficiently sensitive to detect certain effects. Moreover the standard deviations reported on the primary outcome measures demonstrate wide variations pre-and post-intervention. Our study indicates that BCP information needs are very individualized and context-dependent.

Our study had a number of important limitations. It proved difficult to recruit FPs, particularly in the post-implementation phase. The FPs recounted that they may only have a handful of BCPs in their practices at any one time, with breast cancer care being one of many concerns competing for their attention. Further research is required to more robustly sample FP perspectives. Future directions for research include further refinement and evaluation regarding the usability of the SCP components with more BCPs and their FPs. We are making plans to use a human factors approach to evaluate future iterations of the various SCP components [35]. Human factors analysis focuses on “user-centered” design and can be used to evaluate how individuals interact with technological innovations (such as our web tools) as well as their impact on organizational outcomes (e.g. more timely communication between practitioners) [30]. Both cognitive and “sociotechnical” features are explored [30]. While most commonly applied to study technological/computer-based innovations, such approaches could also be applied to the paper-based components of the SCP as well. We wish to examine the impact of all the SCP tools on the quality of patients’ clinical experiences, knowledge, abilities to manage their follow-up care and engagement with their FP. Moreover, we hope to understand the process whereby this intervention can be integrated into the existing infrastructure of care. To accomplish this, we propose to compare the clinical experience of patients within the institution who receive a personalized SCP to those who do not. We agree with Hesse and colleagues (2010), who argue for moving cancer care away from “ a transaction-based approach [to information technology] to one emphasizing long-term support for healing relationships ” and towards user-centered research that explores the meaning that end-users make of various technological and non-technological components to survivorship care [35] p. 81.

## Conclusion

In conclusion, the IOM notes that cancer survivors often feel ‘lost in transition’ to follow-up care, and our findings confirm that BCPs frequently feel ‘lost’ when they transition from specialist treatment to re-enter conventional primary care. Our study also reveals a complex and evolving set of information needs experienced by patients throughout their cancer care journey. Our SCP represents an important first step at our facility for addressing patients’ complex information needs, by tailoring our components to different stages of treatment and life circumstances.

## Abbreviations

BCP: Breast cancer patient; FP: Family physician; IOM: Institute of Medicine; OHCP: Oncology specialist health care providers; SCP: Survivorship care plan.

## Competing interests

The authors declare that they have no competing interests. Funding for this study was generously provided by the Wings of Hope Foundation.

## Authors’ contributions

RQ was the prinicipal investigator for the study and was responsible for the conception, design and overall implementation of the study, as well as designing the components of the survivorship care plan, and contributing to the manuscript. LH assisted in the design of the study, played a leading role in study implementation, data collection and analysis/interpretation, design of the components of the survivorship care plan, and helped to draft the manuscript. NAB assisted with data collection and analysis and interpretation, and helped to draft the manuscript. DD assisted with the design and implementation of the study and contributed to writing the manuscript. FHL contributed to the design of the study and the components of the survivorship care plan, as well as contributing to manuscript writing. EL contributed to the design and evaluation of the survivorship care plan, assisted with the interpretation and analysis, and contributed to drafting the manuscript. BL took a leading role in the design and implementation of the web-based components of the survivorship care plan and contributed to writing the manuscript. KV assisted with the design of the survivorship care plan and assisted with the manuscript writing. JAP led the methodological design of the study, assisted with data analysis and interpretation, assisted in the design of the survivorship care plan, and took the primary responsibility for drafting the manuscript. All authors read and approved the final manuscript.

## Pre-publication history

The pre-publication history for this paper can be accessed here:

http://www.biomedcentral.com/1472-6947/13/76/prepub
